# Medium and Short Wave RF Energy Harvester for Powering Wireless Sensor Networks

**DOI:** 10.3390/s18030768

**Published:** 2018-03-03

**Authors:** Jesus A. Leon-Gil, Agustin Cortes-Loredo, Angel Fabian-Mijangos, Javier J. Martinez-Flores, Marco Tovar-Padilla, M. Antonia Cardona-Castro, Alfredo Morales-Sánchez, Jaime Alvarez-Quintana

**Affiliations:** 1 Advanced Materials Research Center S. C. -Monterrey, Alianza Norte # 202, Autopista Monterrey-Aeropuerto Km.10., C.P. 66600 Apodaca, Nuevo León, Mexico; jesus.leon@cimav.edu.mx (J.A.L.-G.); acl_emc2@hotmail.com (A.C.-L.); angel.mijangos@cimav.edu.mx (A.F.-M.); javier.martinez@cimav.edu.mx (J.J.M.-F.); marco.tovar@cimav.edu.mx (M.T.P.); antonia.cardona@cimav.edu.mx (M.A.C.-C.); alfredo.morales@cimav.edu.mx (A.M.-S.); 2 Genes-Group of Embedded Nanomaterials for Energy Scavenging, CIMAV-Unidad Monterrey, C.P. 66600 Apodaca, Nuevo León, Mexico

**Keywords:** Internet of Things, RF energy scavenging, AM waves detection, full wave Cockcroft–Walton multiplier

## Abstract

Internet of Things (IoT) is an emerging platform in which every day physical objects provided with unique identifiers are connected to the Internet without requiring human interaction. The possibilities of such a connected world enables new forms of automation to make our lives easier and safer. Evidently, in order to keep billions of these communicating devices powered long-term, a self-sustainable operation is a key point for realization of such a complex network. In this sense, energy-harvesting technologies combined with low power consumption ICs eliminate the need for batteries, removing an obstacle to the success of the IoT. In this work, a Radio Frequency (RF) energy harvester tuned at AM broadcast has been developed for low consumption power devices. The AM signals from ambient are detected via a high-performance antenna-free LC circuit with an efficiency of 3.2%. To maximize energy scavenging, the RF-DC conversion stage is based on a full-wave Cockcroft–Walton voltage multiplier (CWVM) with efficiency up to 90%. System performance is evaluated by rating the maximum power delivered into the load via its output impedance, which is around 62 μW, although power level seems to be low, it is able to power up low consumption devices such as Leds, portable calculators and weather monitoring stations.

## 1. Introduction

Along with “zero-power” self-sustainable standalone electronics, emerging platforms such as Internet of Things (IoT), Smart Skins (SS), and Smart Cities (SC) have been designed as networks to interconnect, sense, quickly monitor and easily pinpoint problem areas without requiring human interaction, as well as present the information in an accessible way. Evidently, such an interacting world will need to keep millions and millions of interfaced devices powered long term. A feasible answer to the above-mentioned failings is to harvest electromagnetic-waves energy from external environmental sources which could be used as power supply for battery less operation or even extended battery life in such low power consumption devices [1,2]. 

Although there exists a great interest in RF harvesting from ISM and WLAN bands (900–928 MHz and 2.4/5 GHz respectively) [3,4,5,6,7,8,9,10]; TV (54–890 MHz), FM (88–108 MHz) and AM (530–1700 KHz) bands have recently being explored because they are characterized by covering both urban and non-urban areas because of their continuous emission as commercial bands [11,12]. Besides, compared to commercial bands, ISM and WLAN bands because of their relative high frequency, the distance between the RF source and the harvester dispositive becomes fairly short [13]. Hence, AM band is preferred due to its long-range and low attenuation within building materials like concrete [14]. Likewise, AM electromagnetic wave transmitters and receivers do not need to be in line of sight of each other.

In this work we propose an optimized and portable AM-RF energy harvesting to switch on low energy utilization devices. The high yield of the current AM harvester is based on the development of a high-performance AM resonator which is maximized by optimizing the quality factor Q of the antenna, as well as a stage-optimized Cockcroft–Walton voltage multiplier (CWVM) as AC-DC voltage converter [15].

**Figure 1:**
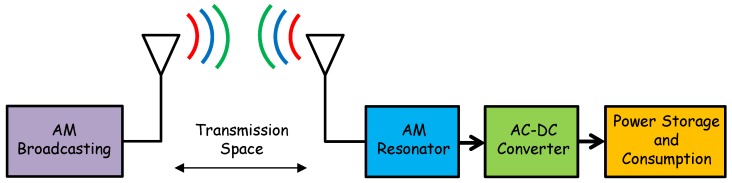
Conceptual platform of an AM-RF energy harvesting system.

The conceptual diagram of an AM RF-energy harvester is shown in Figure 1. Basically, electromagnetic-wave power irradiated by way of AM commercial station is detected by the AM harvester system, and then it is applied to switch on low energy utilization devices. 

Besides, Figure 1 shows a graphical representation of the RF harvester system, which consists of a high-quality factor AM resonator which acts as a high performance receptor, an RF-DC converter, and the power storage and consumption stage. The receptor of electromagnetic waves transduces the AM waves into an AC electrical signal; hence, the AM antenna selects only one signal at a desired frequency. Then, an RF-DC converter transforms the tuned AC signal by the resonator into DC signal, which can finally be stored on an electrical energy storage device such a capacitor or immediately used to switch on a charge. Although all stages in the AM harvester system are important; one of the most relevant ones is AC-DC conversion. The power harvesting device must not only be capable of converting AC signals into DC voltage, but it also must increase the DC signal into an appropriate level, and then supply the maximum power into the charge. This is not an easy task because it depends mainly on the output and input electrical impedance of the converter. Therefore, the key challenges to develop an efficient AM harvester are: (i) efficient yet small antenna to pick up the long wavelengths of the AM electromagnetic waves (177 m–566 m), and (ii) AC-DC converters with proper input and output impedances based on efficient zero threshold diodes for matching to the aforementioned antennas. Hence, because of their long electrical wavelengths, design of compact resonators with satisfactory performance is a very difficult task.

## 2. Portable Medium and Short Wave RF Energy Harvester Design

**Figure 2:**
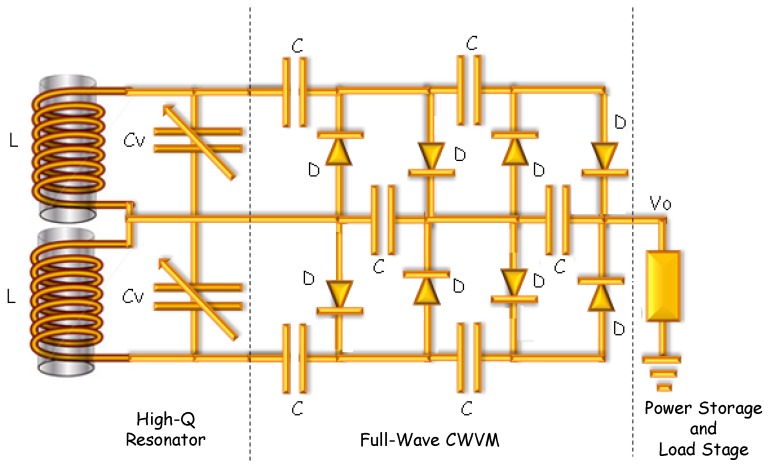
Illustration of the AM-RF harvesting system circuit.

AM stations have some disadvantages compared to other RF sources; for instance, they are limited to transmit power well below 50 kW, and TV stations transmit power up to 1 MW in the USA Besides, due to the long wavelengths, tiny AM antennas broadly go through a very low performance. Nevertheless, despite all those constraints, the model of a high-sensitivity scavenger that is able to harvest very low intensity electromagnetic waves can be reached by designing a high efficiency AM antenna, as well as an optimized AC-DC converter, as it will be explained in the next sections. A detailed electrical circuit of the suggested RF harvester is shown in Figure 2. It is made of the high-Q AM resonator (LC tank circuit), the boost converter (CWVM), and the load which can be either a capacitor for energy storage or a low power consumption device. 

### 2.1. AM Resonator 

**Figure 3:**
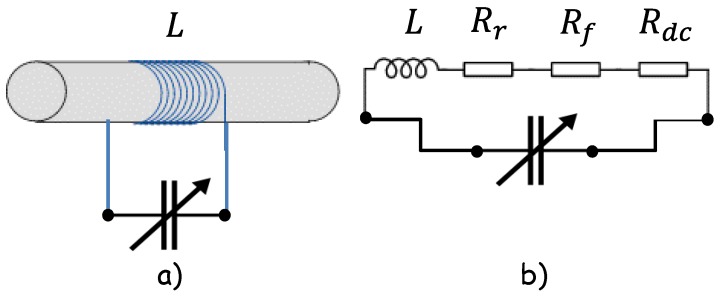
Illustration of (**a**) ferrite rod antenna, and (**b**) equivalent circuit of tuned ferrite rod antenna.

As mentioned previously, because of their long electrical wavelengths, development of compact resonators with appreciable performance is hard work. The crucial parameter is the value of the quality factor (Q) of the inductor (L); as the quality factor of the inductor increases, it also increases the performance of the harvester. Ferrite based antennas are commonly used in AM receivers to enhance the magnetic flux while maintaining a small size; hence, in AM receivers the detected RF signal is further amplified via an RF amplifier, thus a single ferrite core antenna is enough to perform the task efficiently. In general, the efficiency of the ferrite resonator can be improved due to the rise of its quality factor Q well beyond 100. Therefore, ferrite antenna losses because of ferrite core, DC wiring and loop’s radiation resistance must be reduced to improve its efficiency, so the three main parameters are the Q of the tuned circuit, the antenna loss resistance and the radiation resistance. For clarity, tuned ferrite rod antenna and its equivalent circuit including losses due to parasitic resistances is shown in Figure 3a,b respectively.

The resonator radiation *R_r_* resistance is expressed as [16]

(1) $$R_{r}=31200\mu^{2}{\left(\frac{NA}{\lambda }\right)}^{2}$$

where *N* is the number of windings, *μ* is the permeability of the ferrite core, *A* is the cross section of the core, and *λ* is the wavelength of the desired AM-RF wave to be harvested.

For a ferrite rod, the ferrite itself also tends to absorb some of the signal power. This is caused by the requirement that the alternating magnetic field has to “flip” the magnetic alignment of the magnetic domains inside the granular structure of the ferrite. For a ferrite rod, the ferrite loss has an equivalent resistance,

(2) $$R_{f}=\omega \mu_{o}\mu \frac{\mu^{\prime \prime }}{\mu^{\prime }}N^{2}\frac{A}{l}$$

Here, $\mu^{\prime \prime }$ and $\mu^{\prime }$ are the imaginary and real part of the ferrite’s permeability, and *l* the length of the ferrite rod. The quality factor is defined by $Q=\mu^{\prime }/\mu^{\prime \prime }$.

On the other hand, the quality factor *Q* and loss resistance *R_ls_* are related by

(3) $$Q=\frac{\omega L}{R_{ls}}$$

Here, *ω* is the angular frequency of the AM-RF wave detected by the AM resonator, and it is given by $\omega =1/\sqrt{LC}$, where *L* is the inductance of the coil and *C* the capacitance of the variable condenser. 

Here, *L* is given by

(4) $$L=\mu_{o}\mu N^{2}\frac{A}{l}$$

Hence, the total loss resistance *R_ls_* of the ferrite core antenna is defined as [17,18]

(5) $$R_{ls}=R_{dc}+R_{r}+R_{f}$$

where *R_dc_*, *R_r_* y *R_f_* are resistances due to dc wiring, loop’s radiation resistance and for the ferrite rod the extra ‘ferrite’ loss respectively as mentioned previously. Such resistances are related to the loss tangents respectively by

(6) $$R_{ls}=\left(tan\delta_{dc}+tan\delta_{ac}+tan\delta_{f}\right)\omega L$$

And the corresponding loss tangents are given by

(7) $$tan\delta_{dc}=\frac{4\rho_{c}l_{w}10^{9}}{\omega A_{L}Nn\pi d^{2}}$$

(8) $$tan\delta_{ac}=\frac{K_{E}fNnd^{4}}{A_{L}}$$

(9) $$tan\delta_{f}=K$$

where *A_L_* is a geometry coefficient determined by the ratio inductance of the coil and the square of the number of the windings, *L_w_* is the perimeter of each winding, *ρ_c_* is the electrical resistivity of the winding material, *n* and *d* are the number of strands and diameter of each strand of the stranded copper wire, *K_E_* is a geometry proximity effect coefficient, and *K* is a parameter dependent on the properties of the ferrite core. 

**Figure 4:**
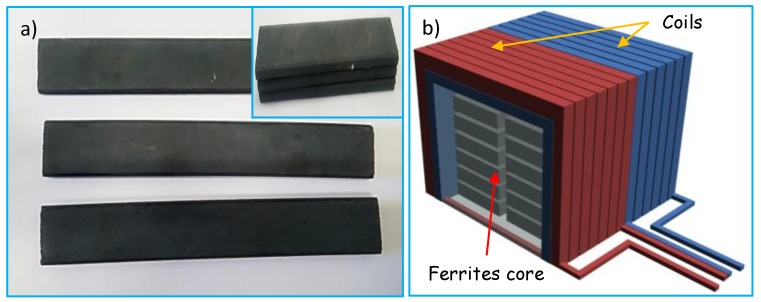
(**a**) Ferrite cores used for antenna coil, inset shows the stacked cores, and (**b**) scheme of the composed ferrite core.

AM antennas based on ferrite cores increase significantly the radiation resistance *R_r_* of the antenna. Therefore, placing the ferrite core in the coil has the effect of raising the radiation resistance by a factor of *μ*^2^, as you can see in Equation (1). Evidently, then the introduction of the ferrite core not only rises the radiation resistance but also reduces the losses due to the resistance of the coil. Moreover, one of the requirements for an efficient ferrite rod antenna is that it should have a *Q* bigger than 100 at the frequencies over which it operates. Thus, according to Equation (3), as *Q* increases *R_ls_* decreases, this reduces the loss resistance and hence the higher performance of the AM harvesting system. Besides, according to Equation (3) an alternative way to increase *Q* is by increasing *L*, in this sense a single core ferrite antenna could be inefficient because of its small cross section *A*. Thus, in the present work in order to enhance *L* via *A* as shown in Equation (4), a ferrite antenna made of a set of stacked planar ferrite cores has been developed. Figure 4a shows the single ferrite cores used for development of the ferrite antenna; the inset shows the stacked cores with the aim of increasing the *L* value of the coil.

Dimensions of a single ferrite core are 100 mm (*l*) × 20 mm (*w*) × 4 mm (*t*), with a value of relative permeability of around 135. By stacking 60 ferrite cores, a composed ferrite core of 100 mm (*l*) × 60 mm (*w*) × 60 mm (*t*) has been developed; in this way, the cross section of the coil has been increased by a factor of 45. Moreover, as you can see in Figure 2, a full wave resonator (double LC tank circuit) has been chosen so as to pick up the total energy present in the amplitude modulated electromagnetic wave. The two coils needed for the double LC tank circuit have been wounded in the same ferrite core by using 22 awg stranded copper wire. Figure 4b shows an illustrative scheme of the composed ferrite core including windings, the value of a single coil is 110 μH, and the value of the two-section variable capacitor varies from 10–100 pF to 365–485 pF. Therefore, the detector is able to tune up frequencies ranging from 590 KHz to 5 MHz, which covers short and medium wave RF spectrum. By using the geometrical and physical parameters of the AM resonator, a value of Q ~ 700 has been estimated, which ensures the high performance of the AM-RF energy harvesting system. 

### 2.2. AC-DC Converter: Cockcroft-Walton Voltage Multiplier

Voltage multipliers are a special type of diode rectifier circuit which can potentially produce an output voltage many times greater than that of the applied input voltage [15]. It has become essential in microwave ovens, strong electric field coils for cathode-ray tubes, electrostatic and high voltage test equipment, etc., where it is necessary to have a very high DC voltage generated from a relatively low AC power supply [19,20,21,22,23,24].

**Figure 5:**
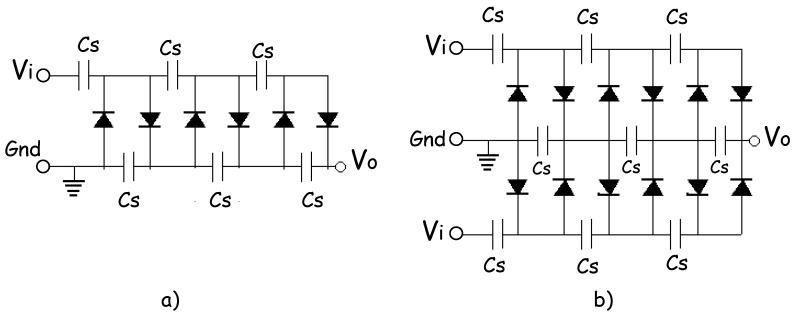
(**a**) circuit for a six-stage conventional CWVM, and (**b**) circuit for a six-stage full wave-CWVM. Here, Cs stands for the series capacitances.

Besides, voltage multipliers are simple circuits made from diodes and capacitors that can increase the input voltage by two, three or *n* times to supply the desired DC voltage to a given load without the need for a heavy step-up transformer. Figure 5a shows the circuit for half-wave conventional CWVM. Theoretically, any desired amount voltage multiplication can be obtained and a cascade of “*N*” doublers, would produce an output voltage of *V*_0_ = 2 *NV_ip_*, where *N* is the total number of stages and *V_p_* the input peak voltage. Nevertheless, with many stages, stray shunt capacitances due to diode junction capacitance permit altering currents to flow in the series capacitances of each stage. In this sense, by increasing the number of stages, the output voltage also is increased. However, it has been previously demonstrated that diode junction capacitance conducts altering currents into stage capacitances, such as current lower output voltage; so there is an optimal number of stages which results in a maximum output power, and this number is around six stages as previously reported [5,12]. 

**Figure 6:**
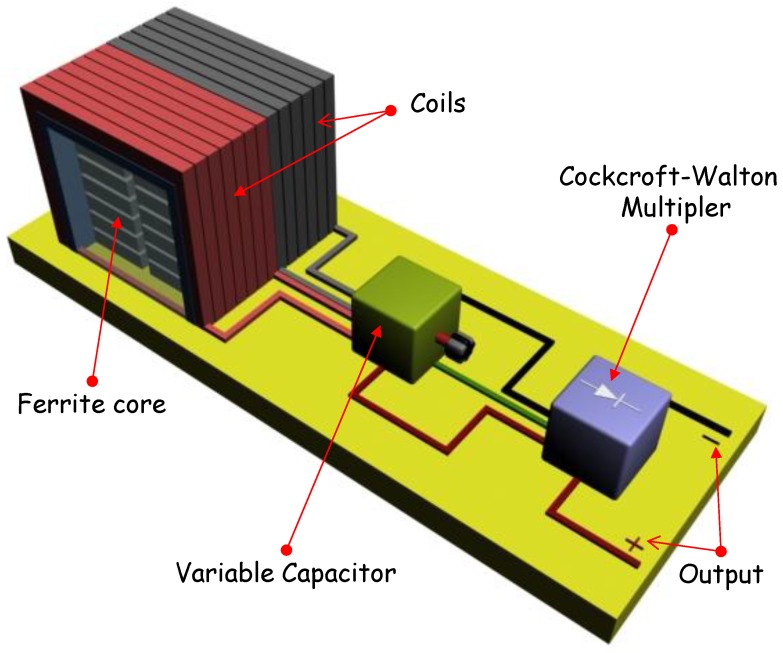
Pictorial image of the AM-RF energy harvesting system.

On the other hand, in AM-RF waves, it can be seen that the envelope of the signal follows the contours of the modulating signal. In fact, there are two enveloping signals being modulated by the modulating signal; this feature leads to an increase in the maximum power which can be obtained from that type of wave. However, in order to get the total power, it is necessary to use a full wave-CWVM instead of a conventional CWVM. Figure 5b shows the circuit for a full wave-CWVM. By using such a circuit, not only the output power is increased, but also the number of charging cycles per second is doubled which cuts down the voltage drop and dramatically reduce ripple factor in the output voltage. According to this, a 6-stage full wave-CWVM has been used in this research in order to increase the power of detected electromagnetic AM wave. The full-wave-CWVM has been based on diodes type BAT85 which present a very low dc forward bias voltage around 200 mV at 25 °C according to datasheet; this striking characteristic ensures low power consumption by the CWVM. Figure 6 shows an illustrative scheme of the implemented AM-RF energy harvesting system; the main parts are also indicated. 

### 2.3. Power Storage and Load Stage

Finally, the output energy from the full wave-CWVM can be stored in an energy storage element, which could be a capacitor or a rechargeable battery. Afterwards, the battery energy storage system can be configured in one of two ways: a power configuration or an energy configuration, depending on their intended application. In our case, the energy was used directly to supply a steady amount of power into the load. In this case, the AM-RF harvester works as a power source; thus, it behaves as an energy source in series with an internal resistance. Therefore, this implies that if we connect a variable load resistance on the power source then the power dissipated by the load will depend on the internal resistance of the power source. Therefore, the load resistance must be matched to the internal resistance of the harvester in order to access to the maximum power given by the AM harvester; this, in accordance to the maximum power transfer theorem. In this sense, previously reported works [12] indicate that frequency of the input RF wave, the number of stages N, the stage capacitances Cs, as well as the rectifier junction capacitance and junction resistance affect the output impedance of the CWVM. For this reason, in the present work the maximum power transferred into load has also been evaluated by implementing the full wave-CWVM using different stage capacitances and different load resistances. In this way, it has been optimized the performance of the AM-RF energy harvesting system.

## 3. Performance of the RF Energy Harvesting System for AM Broadcasting

In order to analyze the functionality of the AM-RF energy harvester, a proof-of-concept device has been implemented according to the design presented in Section 2. The resonator circuit stage plays an important role in the performance of AM harvester, because the amount of the harvested RF energy depends strongly on it. Hence, a resonant circuit with a high Q is preferred in order to get a lot of energy. The advantage of this topology is that it can be easily modified to detect RF energy at different frequencies and power levels in the AM band by simply tuning a variable capacitor.

**Figure 7:**
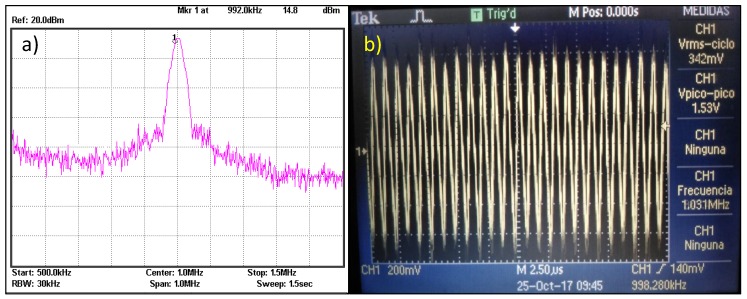
(**a**) RF spectrum of the AM band detected, and (**b**) AM signal detected by the LC circuit is 1.5 V at 1 MHz.

Figure 7a shows the detected RF spectrum; clearly, in the central part of the spectrum appears the 1 MHz signal. Based on this, the AM harvester has been tuned to 1 MHz. Figure 7b presents the signal detected by the resonant circuit; it can be observed a value of 1.5 Vpp at 1 MHz. 

**Figure 8:**
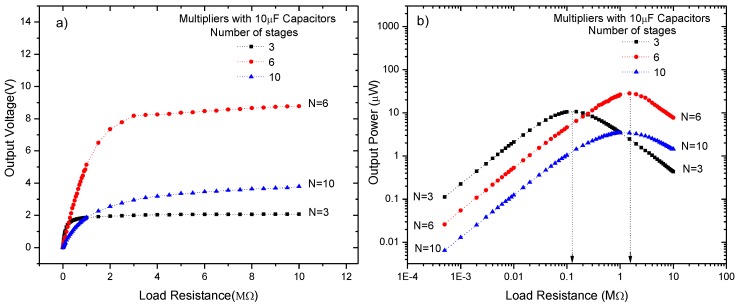
(**a**) Output voltage, and (**b**) output power vs the load resistance for a CWVM with different number of stages. Detected input signal in LC resonator is 1.5 V at 1 MHz.

Figure 8a shows the output voltage vs the load resistance for a conventional CWVMs based on 10 μF stage capacitors, and with different number of stages, *N* = 3, 6 and 10. For all CWVMs it can be observed that as the number of stages increases no necessarily the output voltage increases; for instance, V_out_ (*N* = 6) ˃ V_out_ (*N* = 10). Moreover, Figure 8b shows the power delivered into the load resistance. Evidently, when Z_out_ ≈ R_load_, and *N* = 6, a maximum power is dissipated on the load resistance. Therefore, as the number of stages increases, the output impedance increases. Hence, by modifying *N* the output impedance of the system can be controlled according to the load. 

**Figure 9:**
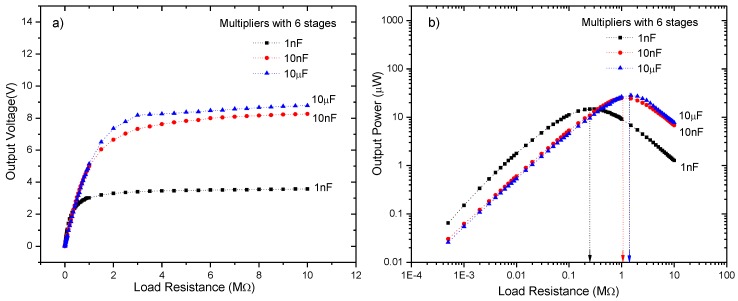
(**a**) Output voltage, and (**b**) output power vs the load resistance for a CWVM with *N* = 6 and with different stage capacitors. Detected input signal in LC resonator is 1.5 V at 1 MHz.

In addition, the output power of the device has been evaluated vs the load resistance and stage capacitors but by fixing the number of stages to *N* = 6. Thus, three CWVMs with identical diodes but different stage capacitors, 1 nF, 10 nF, and 10 μF, has been characterized. Results are shown in Figure 9a. It can be seen that output voltage depends also on the stage capacitors; in fact, as the stage capacitance increases the output voltage increases. Besides, by plotting the output power of the harvester as a function of the load resistance as shown in Figure 9b, it can be observed that there is an optimal capacitance for maximum output power. In this case, a value of 10 μF for stage capacitors delivers the maximum power into the load; thus, capacitance values below and above this value result in a lower output power. Clearly, the experimental output impedance is around 1.5 MΩ for the CWVM with *N* = 6 and C = 10 μF, it means that loads with impedances around 1.5 MΩ will get the maximum power from the AM harvester. 

**Figure 10:**
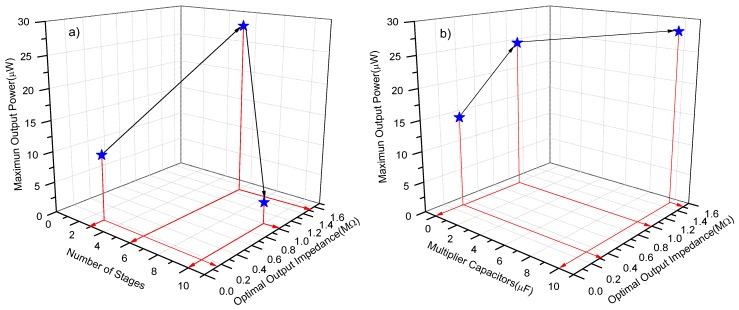
(**a**) Maximum output power vs the number of stages of the CWVM and load resistance, and (**b**) Maximum output power vs of the stage capacitors of the CWVM and load resistance.

In order to clarify the effect of the number of stages and the output impedance on the output power of the harvester, the output power of the RF harvester has been measured as function of such variables. Results are shown in Figure 10a. Evidently, there exists an optimum number of stages which defines a maximum output impedance; as a result, such an optimal point delivers a maximum output power into load. Therefore, an increment in the number of stages does not necessarily increase the output power; however, an increment in stage capacitances not only increases the output impedance, but also the output power, as you can see in Figure 10b. 

**Figure 11:**
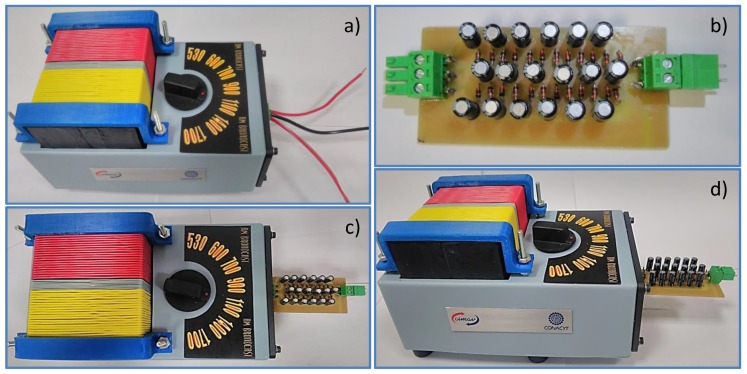
(**a**) AM resonator implemented, (**b**) Full-wave CWVM implemented, (**c**) top view of the AM resonator and full-wave CWVM, and (**d**) lateral view of the AM resonator and full-wave CWVM.

Figure 11a shows an image of the implemented AM-RF harvester system without CWVM. Such a prototype detects RF signals ranging from 540 KHz to 1700 KHz via the tuning capacitor, the signal detected is shown in Figure 7b, and it corresponds to a commercial AM station transmitting at 1000 KHz. The CWVM used in the present prototype is shown in Figure 11b, and it corresponds to a full-wave CWVM. As explained previously, the aim for using a full-wave CWVM is to get the double of the power from the RF signal. Based on the above discussion, in order to obtain the maximum power from the harvester a 6-stages/10 μF dc-dc CWVM with BAT85 rectifiers has been selected. Figure 11c,d show a top and lateral view respectively of the RF detector (AM resonator) and the CWVM once they have been integrated.

**Figure 12:**
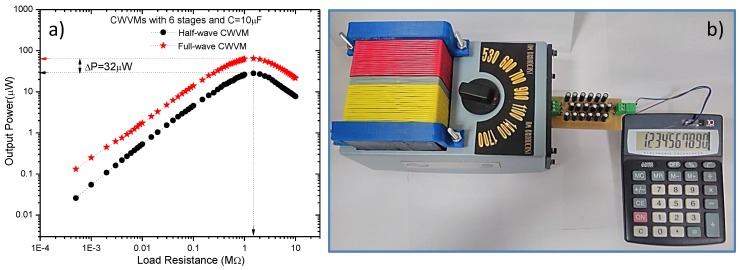
(**a**) Output power for a half-wave and full-wave CWVM, and (**b**) portable electronic calculator running with the AM energy harvester. Detected input signal in LC resonator is 1.5 V at 1 MHz.

The performance of the AM-RF harvester based on a full-wave CWVM is evaluated by way of the maximum output power transferred into the load. To compare the performance, such system is contrasted against a conventional CWVM (half-wave), results are shown in Figure 12a. As expected, the maximum power obtained from a full-wave CWVM is twofold that of the conventional one. 

Figure 12b shows an image of the AM-RF energy harvester connected to a portable electronic calculator. Evidently, the device works properly, it is worth mentioning that prior to this action, the battery was removed from the calculator, thus the device is working only with the RF energy harvested from a commercial AM station located at 2.5 Km. away from the harvester. AM station broadcasting frequency and power is 1 MHz and 10 KW respectively.

On the other hand, it is well known that total system efficiency depends on the efficiency of each of the stages that are part of the system. Thus, each component introduces its own inefficiency to the entire system. Each efficiency is multiplied together to obtain an overall efficiency for the system. In this case, the AM harvester system is mainly composed of three stages; the LC resonator (LC tuned antenna), the Dc-Dc CWVM (boost converter), and the load. So, the overall efficiency of the system is

(10) $$\eta_{total}=\left(\eta_{ant}\right)\left(\eta_{CWVM}\right)\left(\eta_{load}\right)$$

The antenna efficiency is defined as the portion of power that is not absorbed in the antenna structure as ohmic losses. This is characterized by the radiation resistance, *R_r_*, and the ferrite loss resistance, *R_f_*, with which the antenna efficiency, *η_ant_*, is

(11) $$\eta_{ant}=\frac{R_{r}}{R_{r}+R_{f}}$$

Hence, the antenna efficiency can be derived by combining Equations (1) and (2) into Equation (9). Therefore, the estimated antenna efficiency is around 3.2% taking into consideration that we are using a double LC resonator configuration. This value is very superior to the value of $\eta_{ant}=0.04\%$ reported for a single ferrite core LC resonator used as AM-RF energy harvester [25,26].

Besides, the efficiency of a Dc-Dc CWVM can be estimated from

(12) $$\eta_{CWVM}=\frac{P_{L}}{P_{L}+2C_{j}f\left(V_{L}^{2}/N^{2}\right)}$$

where *P_L_* and *V_L_* stands for power and voltage on the load respectively. *N* is the number of stages and *C_j_* and *f* the diode junction capacitance and operating frequency of the CWVM. Evidently, the efficiency depends on the value of the load. In case of the load matching, Equation (12) transforms into

(13) $$\eta_{CWVM}=\frac{P_{Lmax}}{P_{Lmax}+2C_{j}f\left(V_{Lmax}^{2}/N^{2}\right)}$$

By taking the experimental data of *P_Lmax_* and *R_Lmax_* from Figure 12a, *V_Lmax_* can be calculated. Moreover, junction capacitance for BAT85 diode is around 5 pF, operating frequency at 1 MHz, and *N* = 6, and 12 for the half-wave and full-wave CWVMs respectively. Hence, efficiencies $\eta_{hw}=70.6\%$ and $\eta_{fw}=90.6\%$ are estimated for the half-wave and full-wave boost converters respectively. Such efficiency values are competitive with previously reported boost converters based on the discontinuous mode architecture which present an efficiency of 60% [25,26].

Finally, the efficiency of resistive loads is 100% because the total power supplied to the resistive element is dissipated; therefore, based on Equation (10) the overall efficiency of the AM-RF harvester system is 1.13% and 2.9% for the half-wave and full-wave boost converter respectively. Such value could seem to be a low efficiency; however, such efficiencies are 47 and 120 times larger than that of 0.024% estimated for AM-RF energy harvester based on a single ferrite core LC resonator with $\eta_{ant}=0.04\%$, and a discontinuous mode converter with $\eta_{Converter}=60\%$ [25,26]. 

To compare, Table 1 shows the amount of RF energy harvested from different RF power sources at different distances. In general, the power harvested in the present work is very significant, especially if we consider the broadcasting distance as well as the power. For instance, the maximum power harvested by a similar harvester previously reported by Wang et al. [25,26] at a distance of 2.5 km is 0.6 μW, whereas that of the system here developed is 62 μW. Besides, for a harvester with similar power harvested of 60 μW at 4.1 km, the broadcasting power must be as high as 960 KW.

## 4. Conclusions

An AM-RF energy harvester has been developed as a proof-of-concept device so as to power up low power consumption devices such as portable electronic calculators located at 2.5 km away from the AM broadcasting. The maximum RF power harvested is achieved via a full-wave six-stage CWVM based on BAT85 diodes and 10 μF capacitors, and an efficiency $\eta_{fw}=90.6\%$. In this sense, experimental data show that the output impedance of the device depends strongly on the stage capacitors. In fact, as the stage capacitance increases, the output impedance of the harvester also increases. This striking characteristic allows for the control of the output impedance of the harvester system according to the input impedance of the load with the aim of transferring the maximum power into it. In fact, a system with output impedance of 1.5 MΩ delivers a maximum power of 62 μW into an electronic calculator. Hence, it is demonstrated that energy-harvesting technologies combined with low power consumption ICs eliminate the need for batteries, removing an obstacle to the success of the emerging networks such as IoT, SS, and SC. Therefore, it is demonstrated that AM-RF signals can be used as an alternative source of “free energy” to power the next generation of wireless networks.

## Figures and Tables

**Table 1:** Comparison among several RF energy harvesters.

Frequency	Broadcasting Power	Distance	Energy Harvested
1 MHz [This work]	10 KW	2.5 km	62 μW
1.27 MHz [25,26]	50 KW	2.5 km	0.6 μW
902–928 MHz [27]	4 W	15 m	5.5 μW
868 MHZ [28]	1.78 W	25 m	2.3 μW
915 MHz [29]	3 W	11 m	1 μW
674–680 MHz [30]	960 KW	4.1 km	60 μW

## References

[1] Vullers R.J.M., Van Schaijk R., Doms I., Van Hoof C., Mertens R. (2009). Micropower energy harvesting. Solid State Electron..

[2] Harb A. (2011). Energy harvesting: State-of-the-art. Renew. Energy.

[3] Le T., Mayaram K., Fiez T. (2008). Efficient far-field radio frequency energy harvesting for passively powered sensor networks. IEEE J. Solid State Circuits.

[4] Paing T., Shin J., Zane R., Popovic Z. (2008). Resistor emulation approach to low-power RF energy harvesting. IEEE Trans. Power Electron..

[5] Devi K.K., Din N.M.D., Chakrabarty C.K. (2012). Optimization of the voltage doubler stages in an RF-DC convertor module for energy harvesting. Circuits Syst..

[6] Volakis J.L., Olgun U., Chen C.-C. (2012). Design of an efficient ambient WiFi energy harvesting system. IET Microw. Antennas Propag..

[7] Hagerty J., Helmbrecht F., McCalpin W., Zane R., Popovic Z. (2004). Recycling ambient microwave energy with broad-band rectenna arrays. IEEE Trans. Microw. Theory Tech..

[8] Monti G., Congedo F., De Donno D., Tarricone L. (2012). Monopole-based rectenna for microwave energy harvesting of UHF RFID systems. Prog. Electromagn. Res. C.

[9] Russo M., Šolić P., Stella M. (2013). Probabilistic modeling of harvested GSM energy and its application in extending UHF RFID tags reading range. J. Electromagn. Waves Appl..

[10] Jeong T. (2013). Energy-harvesting system design through Bluetooth environment for smart phone. IET Sci. Meas. Technol..

[11] Piñuela M., Mitcheson P.D., Lucyszyn S. (2013). Ambient RF energy harvesting in urban and semi-urban environments. IEEE Trans. Microw. Theory Tech..

[12] Leon-Gil J.A., Perales-Cruz J.C., Licea-Jiménez L., Perez-Garcia S.A., Alvarez-Quintana J. (2015). RF energy scavenging system for DC power from FM broadcasting based on an optimized Cockcroft-Walton voltaje multiplier. J. Electromagn. Waves Appl..

[13] Mikami S., Matsuno T., Miyama M., Kawaguchi H., Yoshimoto M., Ono H. (2006). An energy harvesting wireless-interface SoC for short-range data communication. IEEJ Trans. Electron. Inf. Syst..

[14] Halabe U.B., Maser K., Kausel E. (1989). Propagation Characteristics of Electromagnetic Waves in Concrete.

[15] Cockcroft J.D., Walton E.T. (1932). Experiments with high velocity positive ions. (I) Further developments in the method of obtaining high velocity positive ions. Proc. R. Soc. A Math. Phys. Eng. Sci..

[16] Balanis C. (1997). Antenna Theory: Analysis and Design.

[17] Sazonov E., Li H., Curry D., Pillay P. (2009). Self-powered sensors for monitoring of highway bridges. IEEE Sens. J..

[18] Marian V., Allard B., Vollaire C., Verdier J. (2012). Strategy for Microwave Energy Harvesting from Ambient Field or a Feeding Source. IEEE Trans. Power Electron..

[19] Maksimovic D., Cuk S. (1991). Switching converters with wide DC conversion range. IEEE Trans. Power Electron..

[20] Luo F.L., Ye H. (2003). Positive output super-lift converters. IEEE Trans. Power Electron..

[21] Tseng K.C., Liang T.J. (2004). Novel high-efficiency step-up converter. IEE Proc. Electr. Power Appl..

[22] Inaba C.Y., Konishi Y., Nakaoka M. (2004). High-frequency flyback-type soft-switching PWM DC–DC power converter with energy recovery transformer and auxiliary passive lossless snubbers. IEE Proc. Electr. Power Appl..

[23] Hwang F., Shen Y., Jayaram S.H. (2006). Low-ripple compact high-voltage DC power supply. IEEE Trans. Ind. Appl..

[24] Shenkman A., Berkovich Y., Axelrod B. (2004). Novel AC–DC and DC–DC converters with a diode-capacitor multiplier. IEEE Trans. Aerosp. Electron. Syst..

[25] Wang X., Mortazawi A. (2014). Medium wave energy scavenging for Wireless structural health monitoring sensors. IEEE Trans. Microw. Theory Tech..

[26] Wang X., Mortazawi A. High sensitivity RF energy harvesting from AM broadcasting stations for civilian infrastructure degradation monitoring. Proceedings of the IEEE International Wireless Symposium (IWS).

[27] Kwan J.C., Fapojuwo A.O. (2017). Radio Frequency Energy Harvesting and Data Rate Optimization in Wireless Information and Power Transfer Sensor Networks. IEEE Sens. J..

[28] Stoopman M., Keyrouz S., Visser H.J., Philips K., Serdijn W.A. A self-calibrating RF energy harvester generating 1 V at −26.3 dBm. Proceedings of the IEEE Synposium on VLSI Circuits.

[29] Powercast Documentation. http://Powercastco.com/.

[30] Sample A.P., Parks A.N., Southwood S., Smith J.R. (2013). Wireless Ambient Radio Power. Wirelessly Powered Sensor Networks and Computational RFDI.

